# Forgetting what shouldn't be forgotten: the new normal after the COVID-19 pandemic in Brazil

**DOI:** 10.3389/fpsyg.2024.1362183

**Published:** 2024-07-03

**Authors:** Jéssica Paula Martins, Fernando Augusto Lima Marson

**Affiliations:** ^1^Laboratory of Molecular Biology and Genetics, São Francisco University, São Paulo, Brazil; ^2^Laboratory of Clinical and Molecular Microbiology, São Francisco University, São Paulo, Brazil

**Keywords:** behavior, Brazil, COVID-19, neuroscience, pandemic, psychology, routine, SARS-CoV-2

Dear Editor, the pandemic caused by the severe acute respiratory syndrome coronavirus 2 (SARS-CoV-2) has claimed more than seven million lives around the world since the announcement made by the World Health Organization (WHO) on January 30th, 2020, declaring its beginning (WHO COVID-19 Dashboard, 2024). More than four years have passed and, what was previously considered just hope in the face of an uncertain future, on May 5th, 2023, the end of the global public health emergency against coronavirus disease (COVID)-19 was decreed. This pronouncement came after the WHO Coronavirus Dashboard measured declines in both mortality rates and the burden on health systems. However, even with the end of the emergency, it should be considered that there are still risks of new outbreaks, especially with the increase in underdiagnosis of COVID-19 and the emergence of new genetic variants such as the Omicron ones (Palamim et al., 2023; Colson et al., 2024; Coronavirus Disease Pandemic, 2024).

Since the beginning of the COVID-19 pandemic, numerous countries have adopted measures to contain the spread of SARS-CoV-2. The main strategies to contain the contagion were social distancing, wearing masks, and lockdown, the latter more intensely (Marson and Ortega, 2020; Boschiero et al., 2021; Anderson and Stockman, 2022). However, considering the current Brazilian scenario, it is observed that the behavior of people in a post-pandemic world opens a window to potential forgetfulness of what the disease is capable of, since even with the recommendations of public health specialists, Brazilians presented attitudes that challenged their own survival during the COVID-19 pandemic (Martins et al., 2023).

In view of the lockdown, it is suggested that this condition was not fulfilled by most Brazilians due to the lack of planning by the authorities regarding spending on public policies (Boschiero et al., 2021). It is known that the COVID-19 pandemic has impacted the global economy and accentuated poverty in emerging countries (Coronavirus Disease Pandemic, 2024), while in Brazil it was possible to observe the existing social inequalities throughout the territory, in which the investments offered to promote the confrontation of the pandemic did not reach the entire population (Martins et al., 2023). Financial benefits were created as an interim measure, but many informal workers did not have access to this resource and/or were excluded from the list of beneficiaries. Thus, remaining in confinement was not an option for many Brazilians who needed to somehow provide for their own livelihood.

Regarding the use of masks, it was observed that people were apprehensive at the beginning of the pandemic in the face of the new reality in which the world found itself, as there was a fear of suffering any type of contamination. Speeches and behaviors were guided by concerns about the consequences of COVID-19, especially the possibility of death. So, wearing masks and maintaining social distancing were effective in the beginning. However, over these four years of the COVID-19 pandemic, it has been noted that people no longer adhered to protection recommendations (Anderson and Stockman, 2022). Have they lost their fear of possible contamination?

According to neuroscience, it is suggested that the possibility of people's reduction in maintaining self-care during the COVID-19 pandemic may be related to psychological issues (Roy et al., 2022). That is, when the WHO decreed a state of public emergency at an international level due to the SARS-CoV-2 that spread across all continents, fear dominated people. Being afraid is natural and essential to guarantee survival and when we are exposed to some type of danger, we tend to manifest certain behaviors, such as running away, for example. At the beginning of the pandemic context, we fled the virus by adopting the use of masks, maintaining distance, and lockdowns.

However, the fear did not last long. Have people forgotten everything that has been experienced since 2020? It is observed that, at some point, COVID-19 ceased to be considered dangerous in people's perception. This fact may be related to their own experiences, as the population was being vaccinated, and having family members who had recovered may also have contributed to the idea that COVID-19 did not require all the efforts that were being made (Vann et al., 2022). This fact is justified by the popular belief that everything would be fine, in addition to economic problems and political conflicts that also favored the idea that there was no reason to fear the virus, and that people needed to get back to routine to ensure survival.

Neuroscience states that the human brain has the ability to adapt to new situations, however, the quarantine process has made people less tolerant of isolation. It is suggested that such difficulty may be associated with the mental illnesses that were intensified by the COVID-19 pandemic itself since, during the lockdown, insomnia and anxiety problems were enhanced by the feeling of loneliness (Vann et al., 2022). Mental health was significantly impacted by psychological stress resulting from social isolation and the constant fear of the virus. This scenario increased levels of anxiety and depression, negatively affecting sleep quality, and contributing to a sedentary lifestyle and irregular eating habits (Ashouri et al., 2023; Taheri et al., 2023a; Rassolnia and Nobari, 2024).

A study with 525 students, of which 82.23% were women, revealed that social isolation reduced the practice of physical activities, especially among women. Furthermore, anxiety and depressive symptoms increased by more than 50%, affecting women more intensely compared to men. Surprisingly, those who were physically active before the COVID-19 pandemic and became sedentary after it experienced significantly greater impairment in mental health compared to those who were already sedentary (Rassolnia and Nobari, 2024). Corroborating this information, a study with 581 elite athletes identified that vaccinated athletes showed a greater reduction in physical activity compared to unvaccinated athletes. This phenomenon may be associated with the fact that vaccinated athletes tend to adhere more strictly to social distancing and isolation, motivated by the fear of contracting the virus (Ashouri et al., 2023).

Another study carried out with 1,420 elite and sub-elite athletes (41% women), identified that elite athletes had better psychological conditions compared to sub-elite athletes. Furthermore, elite athletes felt more financially secure. The financial impact can be considered a risk factor for increased stress and symptoms of anxiety and depression in sub-elite athletes, due to the possibility of contract termination. Although high-performance athletes are also exposed to these variables, they do not perceive the financial factor as crucial (Taheri et al., 2023b). Possibly, the same findings about anxiety, depression, and economic barriers described in athletes were common to individuals in the general community, and they were affected in a unique way according to their genotypic, phenotypic, and environmental conditions regarding the impact of COVID-19 at different levels, from individual to community. In this way, the innate and adaptive response to the pandemic was modulated by several factors that, together, resulted in the observed responses to the management of the disease including the adherence to the use of masks, maintaining distance, and lockdowns, also at the individual and community levels.

Confinement tends to reduce motivation and physical performance. During this time, many people may turn to food as a way to deal with their emotions, which provides a false sense of comfort as the relief is only temporary (Taheri et al., 2023a). The relationship between psychological disorders and emotional eating is complex and may lead to an increase in obesity, especially in women. It is estimated that the global prevalence of obesity rose from 11.0 to 25.3% among men and from 15.0 to 42.4% among women during the pandemic (Nour and Altintaş, 2023). A study with 383 participants, of which 63.97% were women, identified a positive correlation between emotional eating and mood disorders. To combat these negative effects, practicing physical activity is essential, as it helps the brain modulate the plasticity of the hippocampus region, contributing to mental and physical health (Taheri et al., 2023a).

However, it was observed that, during the pandemic period, practicing physical activities and maintaining an active life were no longer priorities due to isolation. Have we forgotten the importance of self-care? Amid millions of deaths occurring around the world, was simply being alive enough? Isolation impacted financial security, altered sleep patterns, disrupted bedtimes, and excessive exposure to screens and social media encouraged a sedentary lifestyle (Taheri et al., 2023b). Did mental disorders contribute to the lack of physical activity, or did the decision not to exercise favor the emergence or intensification of emotional problems?

Those who sought mental health professionals may have favored the maintenance of emotional wellbeing and the enhancement of self-esteem during this turbulent period experienced across all continents. These professionals work to promote coping strategies for stressful, limiting, and uncertain situations (Taheri et al., 2023b). Feeling fear is a natural and inherent experience for all human beings, and during the COVID-19 pandemic, this feeling permeated many homes. For psychology, the initial fear of COVID-19 contributed to the population's engagement in adhering to the coping strategies that were proposed, in the hope that soon everything would return to normal. Over the time, the initial fear turned into conformism, as acceptance was the only option left (Vann et al., 2022).

Faced with acceptance, human beings can manifest two aspects: either feeling impotent or acting indifferently, the latter corroborating what was observed in Brazil. Even in the face of a scenario in which there was pain and suffering for the thousands of lives that were claimed by COVID-19, Brazilians, especially the younger ones, began to show an attitude of indifference to the high number of people affected by the SARS-CoV-2. This behavior is explained by psychology as a way for these young people to reaffirm their own autonomy and, as the brain has resources to adapt to adverse situations through neuroplasticity (Wallace and Wallace, 2021) when we talk about the pain and suffering of others, it becomes easier for us to get used to it. On the other hand, a study conducted with 2,067 participants from four countries, including 289 Brazilians, identified significant changes in human behavior during the post-vaccine pandemic period. The most reported changes were distancing from family and friends, with a reduction in the number of visits, followed by a decrease in participation in social events (De Gaetano et al., 2023). However, these data do not represent what was observed in Brazil, as the sample of 289 Brazilians did not reflect the country's real scenario.

Are we used to it? Have we already forgotten what should not be forgotten? Brazil was one of the countries most affected by the COVID-19 pandemic, with damages ranging from economic problems and increased unemployment to crises in health systems and damage to mental health. However, there were no more discussions about COVID-19. Why such a drastic change of behavior of Brazilians in such a short time? It is suggested that several factors may have had an impact on it, from poverty to emotional exhaustion due to the feeling of loneliness. Furthermore, one should take into account the possibility that low adherence to the authorities' recommendations may be associated with low education, since education permeates the understanding of the severity of the virus (Roy et al., 2022).

During the COVID-19 pandemic, the world changed drastically, and we were forced to adapt to countless changes, for example, the impact of increased screen time and reduced social interaction on psychological wellbeing and public health compliance occurred and caused a new style of life. Over time, life adjusted and normality returned, as if that turbulent chapter were just a distant dream. Have we forgotten the marks left by this period? Many Brazilians embodied a line from our national anthem, “giant by its own nature,” demonstrating resilience and strength. However, the return to normality has also highlighted our tendency to underestimate ongoing challenges, as if we were “eternally lying on a splendid cradle,” another reference to the national anthem. Have we truly learned to value collective health and safety?

In addition to possible forgetfulness, there was social pressure exerted by leaders and authorities insisting on the need to return to normality, justifying that the country could not stop. Thus, despite the strengthening of home working, companies reopened, and students returned to schools, all hoping to make up for lost time (Martins et al., 2023). However, this pressure neglected the psychological impacts on many Brazilians. Anxiety, depression, and stress have become constant companions for many, who feel forced to mask their difficulties in the name of supposed normality.

What lessons have we learned from the COVID-19 pandemic? We believed that we would come out of it more human, but the way everything unfolded revealed that we still live in an unequal society. Many people not only lost loved ones but also their jobs, and yet they were pressured to carry on as if nothing had happened. Public policies failed to provide adequate support for mental health, resulting in a scenario that reflected a lack of empathy in the face of the different realities faced during this catastrophic period.

Are we ready to face new challenges? As we sought to resume our routines, we faced other public health crises. Still recovering from a pandemic that took thousands of lives, we encountered new problems: monkeypox and dengue. Monkeypox arrived in Brazil during a time of political and economic crises (Boschiero et al., 2023). Furthermore, this disease carries the stigma of being associated with same-sex relationships, and the president's statements at the time emphasized homophobic behavior, suggesting that only homosexuals were fighting to access vaccines. All of this resulted in distrust in public institutions and healthcare systems, and vaccine hesitancy overwhelmed healthcare services, which were already weakened by the pandemic (Scheffer et al., 2023).

Since 1986, Brazil has faced problems with dengue cases throughout the territory. Despite being a recurring seasonal problem, our public policies still fail to offer effective preventive measures, which favor the contamination of a greater number of people (Mascarenhas et al., 2020). This gap in dengue prevention and control is worrying, as it has persisted for almost 40 years. Have we stopped moving forward? Have we gotten used to this seasonal event? In a post-COVID scenario, this situation could worsen, as resources tend to be directed to fighting pandemics, which could lead to the neglect of other diseases that also require attention.

It is therefore urgent to ask ourselves: are we preventing or just trying to correct the problem? The lack of investment in health promotion and prevention campaigns weakens trust and encourages misinformation. However, it is worth highlighting that, as we seek to restore our routines in a post-COVID scenario, true recovery is not limited to returning to the way we were before. We must value self-care and understand that each person has been affected in a unique way and that the traumas experienced by each individual must be respected.

In the current scenario, the magnitude of the impact of COVID-19 has focused on physical and psychological wellbeing. One of the strategies adopted to avoid chaos is health education. Health bodies have made efforts to transmit relevant information to the population and recommend the most appropriate practices given the situation (Ramalho et al., 2023). However, it is important to reflect on how people perceive and react to these recommendations. While some may see these guidelines as a source of security, others may resist. Health education must be adapted to people's needs, promoting the adoption of healthy behaviors and emotional wellbeing. In brief, the authors discuss the main factors shaping Brazil's ‘new normal' after COVID-19. Figure 1 visually summarizes the findings, making it easier to understand the changes in health and wellbeing.

**Figure 1:**
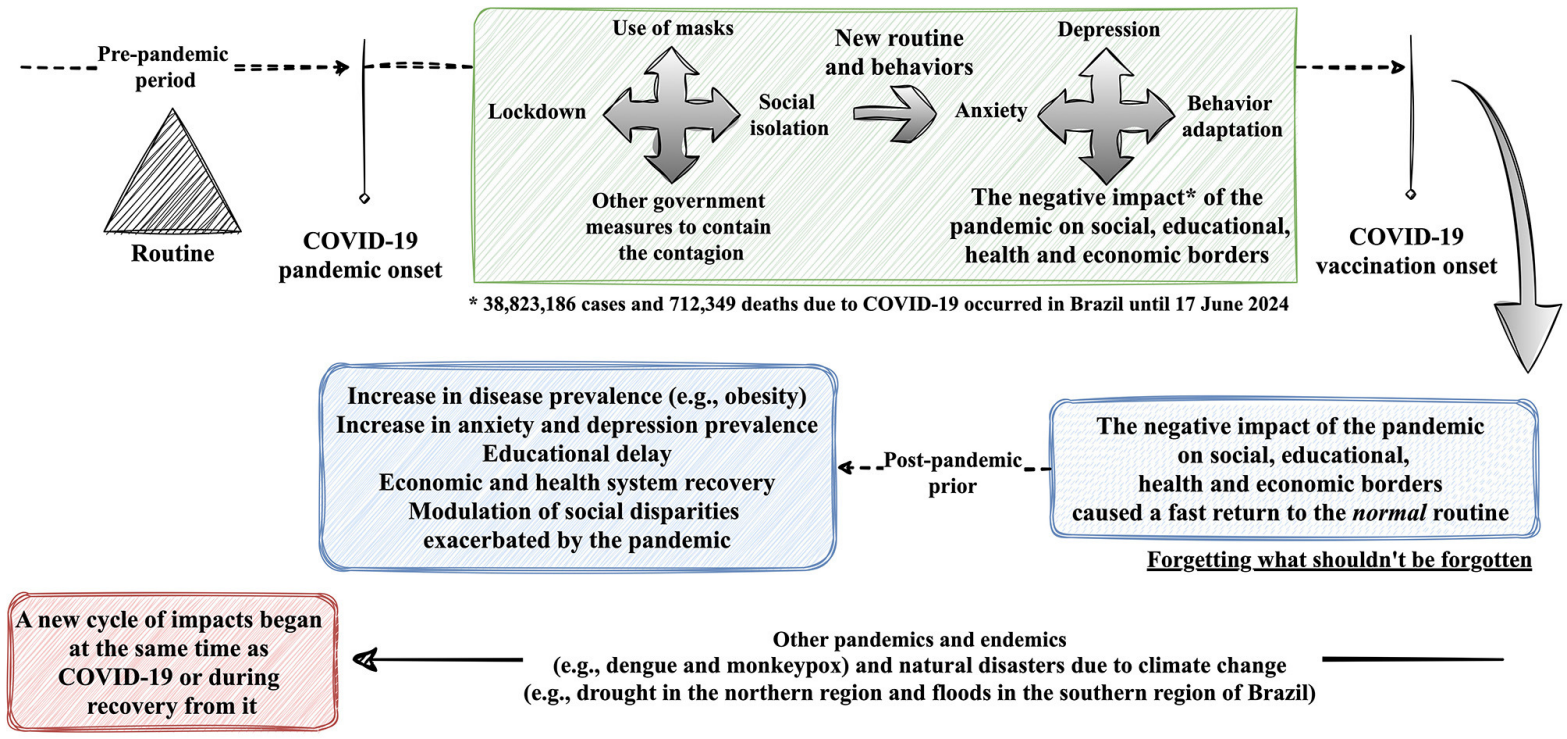
Coronavirus disease (COVID)-19 pandemic in Brazil. Description of the factors associated with the new normal after the COVID-19 pandemic in Brazil, reflecting on forgetting what shouldn't be forgotten.

The consequences of the COVID-19 pandemic have been profound. Although the pandemic was declared over in May 2023, the problems arising from it may persist beyond imagination. In the present study, characterized as an opinion article, a research methodology was not structured, which is considered a limitation. So, as the world recovers, new research must be carried out to understand the impacts of this global crisis. Examining the long-term effects of the COVID-19 pandemic in areas such as mental health, nutrition, physical activity, economics, education, and social inequalities will contribute to strengthening public policies and new health practices that will help face future challenges. As one of Queen's most important songs says—“The Show Must Go On.” The song portrays a powerful expression of resilience and determination in the face of life's adversities. Undoubtedly, in the face of adverse events arising from COVID-19, the show must continue, but we cannot forget each citizen who was lost and each individual who still has physical and emotional consequences due to the disease. Despite the pain, we must express our smile, after all, life is a portrait of the environments we live in behind the curtain of our own life's stage.

## Author contributions

JM: Investigation, Visualization, Writing – original draft, Writing – review & editing. FM: Conceptualization, Investigation, Project administration, Resources, Supervision, Validation, Visualization, Writing – original draft, Writing – review & editing.
